# Recent Advances in CRISPR/Cas9 Delivery Strategies

**DOI:** 10.3390/biom10060839

**Published:** 2020-05-30

**Authors:** Bon Ham Yip

**Affiliations:** Vector Development and Production Laboratory, St. Jude Children’s Research Hospital, Memphis, TN 38105, USA; bonham.yip@stjude.org; Tel.: +1-9018965417

**Keywords:** CRISPR/Cas9, gene editing, delivery, extracellular vesicles, virus-like particles

## Abstract

The clustered regularly interspaced short palindromic repeats (CRISPR)/Cas9 system has revolutionized the field of gene editing. Continuous efforts in developing this technology have enabled efficient in vitro, ex vivo, and in vivo gene editing through a variety of delivery strategies. Viral vectors are commonly used in in vitro, ex vivo, and in vivo delivery systems, but they can cause insertional mutagenesis, have limited cloning capacity, and/or elicit immunologic responses. Physical delivery methods are largely restricted to in vitro and ex vivo systems, whereas chemical delivery methods require extensive optimization to improve their efficiency for in vivo gene editing. Achieving a safe and efficient in vivo delivery system for CRISPR/Cas9 remains the most challenging aspect of gene editing. Recently, extracellular vesicle-based systems were reported in various studies to deliver Cas9 in vitro and in vivo. In comparison with other methods, extracellular vesicles offer a safe, transient, and cost-effective yet efficient platform for delivery, indicating their potential for Cas9 delivery in clinical trials. In this review, we first discuss the pros and cons of different Cas9 delivery strategies. We then specifically review the development of extracellular vesicle-mediated gene editing and highlight the strengths and weaknesses of this technology.

## 1. Introduction

Clustered regularly interspaced short palindromic repeats (CRISPR) were first discovered as an adaptive immune system effector in prokaryotes [1]. CRISPR comprises a group of small DNA sequences found in the genomes of prokaryotes that were acquired from previous infections by bacteriophages [1]. It offers a defense mechanism for prokaryotes to fight against reinfection by similar bacteriophages. Subsequent development of this technology into a gene-editing tool in eukaryotic cells [2,3] enabled the application of gene editing for human diseases. The CRISPR/Cas9 system is composed of a target-specific single guide RNA (sgRNA) and a Cas9 endonuclease. A target-specific sgRNA, formed by the fusion of a CRISPR RNA (crRNA) and a transactivating CRISPR RNA, directs the Cas9 protein to a target site for cleavage, creating a double-strand break (DSB). Because target recognition is based on RNA–DNA interactions, CRISPR/Cas9 has the advantages of the easy design of genomic targets and multiplexing over that of zinc finger nucleases (ZFNs) and transcription activator-like effector nucleases (TALENs). In contrast with ZFNs and TALENs, which require laborious protein engineering steps for each new editing target, the Cas9 nuclease simply requires a target-specific sgRNA for each editing target.

The target specificity of Cas9 is determined by the spacer sequence of crRNAs (~20 nucleotides) and adjacent protospacer adjacent motifs (PAMs) [4]. More precisely, the seed sequence located in the 3′ end of the spacer sequence (10–12 base pairs adjacent to the PAM) is critical for correct targeting [4]. Cas9 will cleave only when sufficient homology is present between the seed region and the target DNA. However, off-target cleavage occurs when DNA sequences contain a few mismatches but share some homology with the seed region of the sgRNA [4]. Research efforts are focused on minimizing the off-target effects associated with CRISPR/Cas9. For example, truncated gRNAs (< 20 nucleotides) reportedly reduce off-target effects without affecting on-target genome editing [5]. Moreover, gene editing with a Cas9 nickase (a mutant that creates a single-strand break in DNA) and two sgRNAs, each cleaving at different sites of the target, dramatically reduces off-target effects [6]. The delivery of a Cas9 ribonucleoprotein (RNP) complex into cells produces fewer off-target effects than does the delivery of DNA plasmids expressing Cas9 and sgRNA [7,8]. Because the Cas9 protein is short-lived and is able to immediately cleave the target DNA, Cas9 RNP delivery mitigates the propensity of Cas9-induced off-target effects.

**Figure 1:**
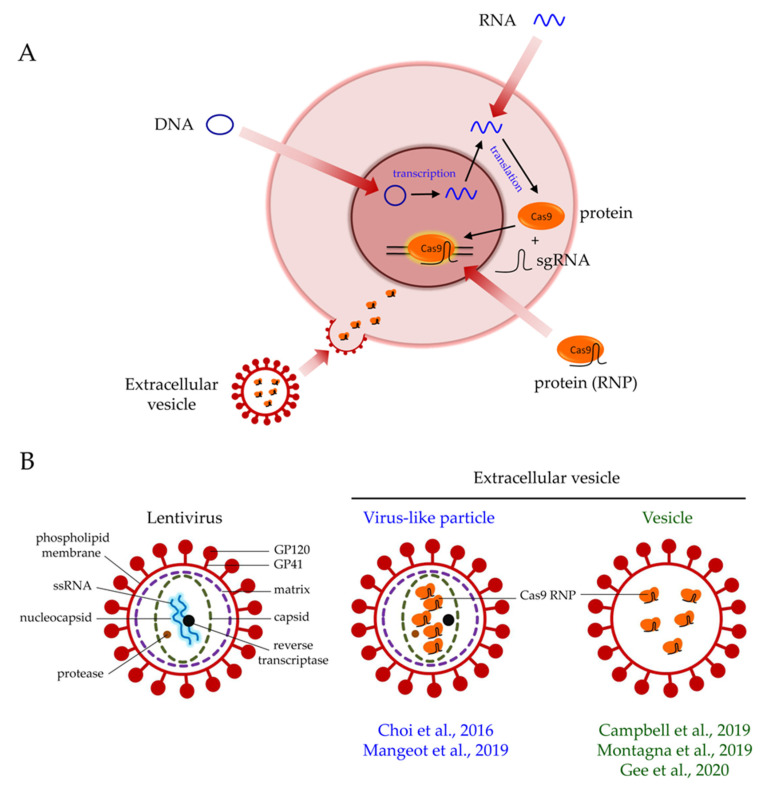
Extracellular vesicle-mediated delivery of Cas9 ribonucleoproteins (RNPs). (**A**) Cas9 can be delivered in the forms of DNA, mRNA, or protein. The protein format enables the immediate action of Cas9 when it is in the nucleus. The transduction of extracellular vesicles releases pre-loaded Cas9 RNPs into cells for efficient gene editing. (**B**) Structural differences between a lentivirus, a virus-like particle, and a vesicle.

The delivery of Cas9 into cells is an important consideration in gene editing. Cas9 can be delivered in the forms of DNA, mRNA, or protein (Figure 1A). Each format has pros and cons. The delivery of Cas9 in the form of plasmid DNA offers a cost-effective option. Only a standard laboratory set-up is required for plasmid preparation. Plasmid DNA-driven Cas9 expression also yields a longer expression time in cells, which may be advantageous if sustained expression is required for editing. However, because transcription and translation are required for the synthesis of the Cas9 protein (Figure 1A), the plasmid DNA format has the slowest onset of editing when compared with that of mRNA and protein. Sustained expression of Cas9 in cells also increases the chance of off-target effects [9]. Furthermore, plasmid DNA poses a risk of insertional mutagenesis [10].

The delivery of Cas9 by mRNA enables the faster onset of gene editing than that by plasmid DNA because transcription is not required anymore (Figure 1A). Because mRNA is highly unstable and prone to degradation by RNases, this format only permits transient Cas9 expression. Chemical modifications of mRNA are available to enhance its stability after delivery [11]. Although transient Cas9 expression may compromise gene editing efficiency, it also reduces the chance of off-target effects [9]. 

The delivery of Cas9 via protein enables immediate gene editing in the nucleus (Figure 1A), resulting in higher gene editing efficiency than that of DNA and mRNA [12]. However, the protein delivery of Cas9 in cells is the most transient of the formats, but the chance of off-target effects is also minimal [12]. The cost of protein delivery is also higher than that of DNA and mRNA delivery. Importantly, delivering the Cas9 protein, which is of bacterial origin, into cells may induce the carryover of bacterial endotoxin and trigger serious immunologic responses. This aspect is a key safety concern of using Cas9 in clinical trials [13]. 

The first clinical trial using CRISPR/Cas9 technology was approved in 2016 [14]. In that trial, the CRISPR/Cas9-mediated knockout of *PD1* was performed in patient blood cells to reactivate T cells for the treatment of lung cancer [14]. Since then, researchers began investigating the potential of using CRISPR/Cas9 for the treatment of other diseases in clinical trials. At the time of writing this review, ClinicalTrials.gov (https://clinicaltrials.gov/) has listed more than 20 clinical trials using CRISPR/Cas9 to treat solid tumors, hematologic malignancies, and genetic disorders. 

To date, many strategies are available for Cas9 delivery. These can be classified into viral and nonviral vector-based approaches. In general, nonviral vector-based approaches include physical and chemical methods. Because extracellular vesicles (EVs) resemble viruses lacking genomes (Figure 1B), the EV-based delivery method represents a compromise between viral and nonviral delivery approaches and possesses the strengths of both approaches (Table 1). Understanding the pros and cons of each delivery strategy is paramount to choosing the most appropriate delivery method for specific applications. In clinical trial settings, stringent safety requirements should be considered, in addition to delivery and editing efficiencies. In this review, we first discuss the advances in CRISPR/Cas9 gene editing and the various Cas9 delivery strategies available today. Researchers are developing and optimizing novel strategies to improve the safety and efficiency profiles of Cas9 delivery. We also discuss the development of EVs for Cas9 delivery and the potential of this strategy for achieving safe and efficient gene editing. 

## 2. Advances in CRISPR/Cas9 Gene Editing

Researchers have been diversifying and optimizing CRISPR/Cas9 applications to achieve various types of editing in the genome. By exploiting the right DNA repair pathway, CRISPR/Cas9 can accomplish gene disruption, deletions, knockins, or targeted editing. The non-homologous end joining (NHEJ) DNA repair pathway is active in any phase of the cell cycle, with increased activity as cells progress from the G1 to G2/M phase [15]. Although the efficiency of NHEJ is high, it is highly error-prone [16]. It frequently introduces insertions or deletions at DSBs, resulting in frameshift mutations that disrupt gene products [16]. Therefore, the NHEJ pathway is commonly used for generating gene knockouts [17]. A complete gene deletion can also be achieved if two sgRNAs are used, with one targeting the start and the other targeting the end of a gene, to generate two DSBs. 

In contrast, the homology-directed repair (HDR) pathway is primarily used to generate gene knockins and for targeted gene editing [17]. The HDR pathway is a more precise pathway than NHEJ [17]. To repair DSBs, HDR requires a donor template, comprising the knockin sequence flanked by 5′ and 3′ homology arms containing homologous sequences to both sides of the DSBs. Although the HDR pathway is less error-prone than NHEJ, it has a much lower efficiency than does NHEJ [17]. Unlike NHEJ, which is active in any phases of the cell cycle, the HDR pathway is active only in the S and G2/M phases [18]. This limits HDR-mediated gene editing to actively dividing cells, restricting its application in the clinical setting, as stem cells are maintained in a quiescent state [19]. 

The recent discovery of homology-independent targeted integration (HITI) enabled gene knockin generation in both dividing and nondividing cells via NHEJ [20]. When a desired transgene is flanked by the Cas9/gRNA target sequence in a donor vector, the Cas9 cleavage of the donor vector and the genome occurs simultaneously. The DSBs generated in both the vector and the genome trigger the NHEJ repair pathway, which integrates the desired transgene into the genome. Because NHEJ is active in all cell cycle phases [15], HITI is a more efficient approach to generate gene knockins than is HDR. However, several challenges must be addressed before HITI can be used in the clinical setting. The knockin efficiency of HITI is currently less than 5% in nondividing cells in most cases [21], and the potential off-target effects of Cas9 may lead to the integration of transgenes at off-target locations. Nevertheless, these off-target effects can be minimized by the stringent selection of Cas9/gRNA target sequences and the use of a high-fidelity version of Cas9 nuclease [22,23]. 

Microhomology-mediated end joining (MMEJ) is an alternative end-joining pathway exploited in CRISPR/Cas9 gene editing [24]. MMEJ is active when microhomology (5–25 bp) is present upstream and downstream of DSBs. This allows the annealing of two microhomology sequences, resulting in the deletion of the intervening sequence [25]. Recently, Nakade et al. developed an MMEJ-based method, termed precise integration into target chromosome (PITCh), to achieve the targeted knockin of transgenes [24]. Cleavage by Cas9 at the PITCh donor vector and the genome exposes their microhomology sequences, which trigger the MMEJ-mediated integration of transgenes into the genome at the DSBs [24]. MMEJ is active during the M and early S phase, when HDR is inactive [26]. Importantly, MMEJ is two to three times more efficient than is HDR at achieving the targeted knockin of transgenes [24]. 

Irrespective of which DNA repair pathway is employed, the generation of DSBs after the Cas9 cleavage of the genome poses a safety concern about genotoxicity [27]. A base-editing strategy that bypasses DNA cleavage has therefore revolutionized the gene editing of point mutations [28,29]. The fusion of a catalytically dead mutant Cas9 (dCas9) protein to cytidine deaminase mediates the conversion of C > T or G > A [28], providing a promising way to correct C or G bases in the genome [27]. However, a lack of deaminases for A or T limited the application of this technology. Approximately 1 year later, however, adenosine deaminase was synthesized via the protein evolution and engineering of a tRNA adenosine deaminase [29]. Adenosine deaminase was subsequently reported to mediate the conversion of A > G or T > C in the genome [29]. Therefore, base editing by the fusion of dCas9 to cytidine deaminase or adenosine deaminase may lead to a safe and efficient approach to editing point mutations. 

Although base editing can mediate the conversion of the four transition mutations, it cannot convert transversion mutations. Recently, Anzalone et al. reported a versatile prime-editing strategy that can achieve targeted insertions, deletions, and conversions of all 12 combinations of point mutations without the need for a donor template [30]. Prime editing requires two components: Cas9 nickase and a prime editing guide RNA (pegRNA) [30]. The pegRNA is an extended version of sgRNA, containing a primer binding site to permit the hybridization of the 3′ end of the nicked genomic DNA and a reverse transcriptase (RT) template containing the desired edit to provide a template for the synthesis of the edited information [30]. The catalytically impaired Cas9 nickase is coupled to an RT and introduces a single-strand nick to genomic DNA to facilitate the binding of the 3′ end nick to the primer binding site of the pegRNA [30]. The RT therefore reverse transcribes the sequence information, including the edit from the RT template to DNA [30]. Currently, prime editing appears to be superior to other editing strategies in terms of its efficiency, genotoxicity, and versatility in gene editing [30]. Nevertheless, further investigation of this strategy in more cell types and the optimization of the delivery strategy is warranted. 

## 3. Common CRISPR/Cas9 Delivery Strategies

The delivery of the CRISPR/Cas9 system for efficient gene editing is challenging. The Cas9 protein has a molecular weight of approximately 160 kDa [31], and after forming an RNP complex, the long phosphate backbone of the sgRNA imparts a net negative charge to the complex [32]. Both of these properties make it difficult for the Cas9 RNP to cross the cell membrane. Moreover, once inside cells, both the Cas9 protein and sgRNA must survive the degradation processes in the cell and translocate into the nucleus to enable gene editing. Therefore, choosing an appropriate delivery strategy for the CRISPR/Cas9 system is critical to achieving efficient and precise gene editing. If it is to be used in clinical settings, the safety profile must also be considered to avoid or minimize insertional mutagenesis. To date, the delivery strategies for CRISPR/Cas9 can be broadly classified into viral or nonviral approaches, depending on whether viral transduction is used. The nonviral approach includes various physical and chemical delivery strategies. Each of these methods has its own pros and cons that should be considered for each gene editing application (Table 1). 

### 3.1. Viral Vectors

Adeno-associated viruses (AAVs) are a common viral vector used for gene delivery. The unique properties of AAVs (i.e., replication-defective, nonintegrating into the genome, and low immunogenicity in humans) have triggered huge interest in their potential as delivery vehicles, especially for in vivo applications [33,34]. After transduction, the AAV genomes remain episomal in the nucleus, which are gradually diluted by cell division. Therefore, the episomal delivery of transgenes by AAV provides a safe option to transiently express genes [35]. 

CRISPR/Cas9 can be delivered by AAVs in two ways: First, AAVs can serve as a vehicle to deliver Cas9, sgRNAs, and/or donor templates into cells by transduction [36]. Indeed, AAVs not only enable in vivo CRISPR/Cas9 genome editing but are also useful in in vitro applications, especially when genome integration is a safety concern and electroporation is not an option for the cell type of interest. However, AAV vectors are limited by their low cloning capacity (< 4.7 kb). A study of metabolic liver disease in mice used two separate AAV vectors, one expressing Cas9 and the other expressing an sgRNA and a donor DNA sequence, to achieve a gene editing event [36]. The delivery of the commonly used *Streptococcus pyogenes* Cas9 by AAVs is challenging because of its large size (~4.2 kb). A smaller strain of Cas9 from *Staphylococcus aureus* (SaCas9; ~3.15 kb) is a more feasible option [37]. However, SaCas9 is restricted by the availability of suitable PAM sequences for targeting [38]. Second, AAV vectors can be used as a donor template for gene knockin through the HDR pathway [39,40]. The knockin efficiency of AAV donor templates is higher than that for nonviral targeting methods [39]. Similarly, the limited cloning capacity of recombinant AAVs can be circumvented by splitting large transgenes into two separate AAV vectors to enable sequential homologous recombination [40]. Another disadvantage of AAVs is their low efficiency in gene targeting. Specific homologous recombination only occurs in ~0.1% to 1% of the total cell population under optimal conditions [41]. Currently, the AAV-based gene editing trials registered on ClinicalTrials.gov only use ZFNs to insert a corrective copy of the gene into the genomes of patients with hemophilia B [42] or mucopolysaccharidosis types I and II [43]. Because AAV-based delivery is expected to become increasingly popular, clinical trials with AAV-based CRISPR/Cas9 gene editing may be forthcoming. 

Lentiviruses (LVs) are another viral vector used for CRISPR/Cas9 delivery. LV vectors have a more generous cloning capacity (< 8 kb) than do AAV vectors, which enables the cloning of both Cas9 and sgRNA into a single LV vector. The production of LVs is also less laborious than that of AAVs. The LV transduction process is highly efficient in a wide variety of cell types in both dividing and nondividing cells [44]. These advantages indicate that LV vectors are an optimal vehicle for delivery in vitro and ex vivo [44]. However, random integration into host cell genomes is the biggest challenge associated with LV systems. The integration of LVs in the vicinity of oncogenes may lead to their activation, resulting in tumorigenesis [45]. This precludes the LV-mediated delivery of CRISPR/Cas9 for in vivo gene editing in clinical trials [46]. Indeed, several tragedies in clinical trials were reported due to insertional mutations introduced by retroviruses [47,48,49,50], indicating the potential danger of using LVs in patients. The development of integration-defective lentiviruses with plasmids expressing mutant integrase may increase the safety of LV transduction [51]. Nevertheless, a variable level of background integration occurs and appears to be unavoidable [52,53]. 

Adenoviruses (AVs) are widely used in clinical trials for gene delivery [54]. AVs transduce both dividing and nondividing cells and, most importantly, do not integrate into host cell genomes [54]. The major challenge of using AVs for delivery is that they trigger a high level of innate immune responses in host cells, resulting in the inflammation of tissues and subsequent removal of AV vectors [55]. The production of AVs is also laborious [56], which limits the application and efficiency of this strategy. 

The efficient delivery of CRISPR/Cas9 by viral vectors generally results in a higher percentage of editing than by other methods. Although this is advantageous in most cases, in certain disease conditions, such as retinal diseases and spinal cord injuries, a modest level of editing or reprogramming in a fraction of the cells can achieve therapeutic effects [57,58]. Over-editing, therefore, may create safety issues in these scenarios. The efficiency of gene editing required should be considered based on each disease condition.

### 3.2. Nonviral Physical Methods

Microinjection is the physical method of injecting Cas9 and sgRNAs directly into cells with a microscope and needle. Because the needle pierces through the cell membrane to directly deliver the cargoes into the nucleus, the molecular weight of Cas9, which is usually an issue in viral vector-mediated delivery due to its limited cloning capacity, is not an obstacle in microinjection. Moreover, manual injection enables the controlled dosing of cargoes into cells. However, microinjection is laborious and technically challenging, rendering this technique low throughput. Furthermore, the requirement of a microscope for injection excludes this technique from in vivo patient work. Indeed, most microinjection applications are used in animal zygotes for the generation of transgenic animal models [59,60,61]. 

Electroporation is a popular physical delivery method. It applies pulses of electrical currents to stimulate the transient opening of pores in cell membranes, permitting the delivery of cargoes into cells. Electroporation is commonly used in in vitro and ex vivo gene editing because it efficiently delivers cargoes into a wide variety of cell types. This is advantageous over standard transfection methods, which are usually hampered in difficult-to-transfect cell types, such as primary cells. Indeed, electroporation-mediated ex vivo gene editing fostered the development of stem cell therapies, especially for the treatment of hematologic malignancies [62,63]. Patient hematopoietic stem/progenitor cells after ex vivo modification are transplanted back into patients for treatment [63]. Although in vivo electroporators are currently available and are reported to successfully accomplish gene editing in animals [64,65,66], the application of electroporation in patients for in vivo gene editing is still not generally feasible. Moreover, the cost associated with electroporation-mediated gene editing is usually high because the extensive optimization of Cas9-to-sgRNA ratios and specific electroporation conditions for each cell type are required. Importantly, the strong electrical current generated by electroporation results in a high percentage of cell death, indicating that this method may not be suitable for stress-sensitive cell types. 

### 3.3. Nonviral Chemical Methods

Lipid-based nanoparticles (LNPs) are commonly used for nucleic acid delivery [67]. Liposomes are spherical structures composed of lipid bilayers formed in aqueous solutions. Because both nucleic acids and cell membranes are negatively charged, the repulsion between them prevents the entry of nucleic acids into cells. The encapsulation of negatively charged nucleic acids into positively charged liposomes thereby facilitates the fusion of the complexes across cell membranes into cells [67]. The CRISPR/Cas9 system can be delivered in the format of DNA (“all-in-one” plasmid), mRNA (Cas9 and sgRNA), or protein (RNP). Currently, the Lipofectamine reagent is the most popular choice for LNP formation. The successful delivery of the CRISPR/Cas9 system with Lipofectamine transfection in vitro and in vivo for gene editing has been demonstrated [12,68,69,70,71]. Non-lipid polymeric reagents, such as polyethylenimine and poly-L-lysine, are also commonly used to generate nanoparticles for the delivery of CRISPR/Cas9 cargoes [72,73]. Similarly, polymeric reagents mediate the encapsulation of CRISPR/Cas9 cargoes into positively charged complexes to enable endocytosis into cells [74]. Because viruses are not involved in nanoparticle-mediated delivery, this method is a safer alternative. Moreover, LNPs do not exert stress on cells to the same extent as electroporation. Consequently, this delivery system is approved by the US Food and Drug Administration for drug delivery [75]. Indeed, both lipid- and polymer-based reagents were used to deliver CRISPR/Cas9 in clinical trials for the treatment of various diseases [10]. Nevertheless, the efficiency of this strategy, which relies solely on the endosomal pathway, is low when compared to that of viral transduction and electroporation. For example, chemical transfection methods result in less than 10% of eGFP expression in human embryonic stem cells. Transgene expression also decreases over time after each cell division [76,77]. This limitation restricts the application of this strategy to certain cell types. 

Cell-penetrating peptides (CPPs) are short peptides with an intrinsic ability to translocate across cell membranes. They have been exploited to facilitate the delivery of a variety of cargoes into cells [78,79,80]. CPPs can be conjugated to Cas9 and sgRNAs separately [8,81] or, in most cases, conjugated only to Cas9 followed by complexing with sgRNAs to form RNPs before delivery [81,82]. CPPs offer an ostensibly safer option for Cas9 RNP delivery because random integration and insertional mutagenesis are not factors. However, CPP-based delivery is inefficient when compared to that of viral and physical methods, resulting in a low percentage of gene editing [83]. Although CPP-based deliveries work well in vitro and ex vivo [8,78,79,80,82], the involvement of multiple parameters at different stages (CPPs, Cas9, sgRNA, and each cell type) introduces variability and requires extensive optimization. Hence, CPP-based delivery is not ideal for achieving efficient in vivo gene editing. 

Gold nanoparticles (AuNPs) can efficiently deliver Cas9 RNPs for gene editing [84,85]. Being chemically inert [86], AuNPs do not trigger an immune response after delivery [85], which increases their safety profile. In the CRISPR-Gold system developed by Lee et al., AuNPs (15 nm) are first conjugated to 5′ thiol modified single-stranded DNA sequences hybridized to single-stranded donor DNA. This is followed by the loading of Cas9 RNPs to donor DNA and then coating with whole particles of silicate and the polymer PAsp(DET) [85]. CRISPR-Gold was demonstrated to induce HDR in cell lines and primary cells with approximately 4% efficiency [85]. These results indicate that CRISPR-Gold is more efficient than Lipofectamine transfection or nucleofection for inducing HDR in vitro [85]. Moreover, intramuscular injection of CRISPR-Gold in mice induced HDR to correct a point mutation in the dystrophin gene in vivo at approximately 5% efficiency [85]. Although additional research is required to improve the editing efficiency of this technique, it provides a safer alternative to viral approaches for HDR-mediated gene editing. 

## 4. Delivery of CRISPR/Cas9 Gene Editing by Extracellular Vesicles

Viral vector-based delivery is highly efficient, but LVs carry the risk of insertional mutagenesis and AAVs have a limited cloning capacity for cargoes (Table 1). Physical and chemical methods, however, are generally not effective in vivo, which limits their usage in clinical trials (Table 1). Recently, several studies reported the efficient delivery of Cas9 RNPs in vitro and in vivo by EVs [87,88,89,90,91], indicating the potential of using this platform in clinical settings. 

The formation of EVs is based on the expression and self-assembly of the viral envelope and/or viral structural proteins (Figure 1B) [90,92,93]. The term virus-like particles (VLPs) is used if both the viral envelope and viral structural proteins are assembled into EVs. The protein of interest is fused to the Gag polyprotein so that they can be incorporated simultaneously into a particle. During the viral maturation process, the expression of the protease from *Pol* mediates cleavage along the Gag polyprotein, releasing the protein of interest for delivery [94]. In contrast, the term vesicles should be used when only the viral envelope proteins, such as the envelope glycoprotein of the vesicular stomatitis virus (VSV-G), are assembled into a particle. Vesicles contain no or minimal viral structural proteins. The formation of vesicles is not dependent on the Gag polyprotein and protease cleavage. Vesicles are formed from the budding of the cell membrane when viral envelope proteins are overexpressed [90,92]. Nevertheless, in contrast with LVs, EVs do not contain any viral genome (Figure 1B). Therefore, EVs are not integrated into host genomes and do not replicate [95]. This superior safety feature of EVs renders them a safe version of viral delivery. Moreover, the transient exposure of Cas9 by EVs in cells greatly reduces the chance of off-target effects due to long-term Cas9 expression [9]. The production of EVs is also simple and cost-effective, because only the standard transfection of plasmids into packaging cells is required. Indeed, VLPs have been exploited extensively in vaccine development [95]. VLP-based vaccines deliver antigenic proteins into hosts to provoke an immune response, which is much safer than the traditional approach of using attenuated viruses [95,96]. The delivery of other proteins, including fluorophores, Cre recombinase, and human caspase 8 by VLPs has also been demonstrated [97]. Thus, current research efforts are shifting to Cas9 protein delivery with EVs [87,88,89,90,91]. 

To reduce the off-target effects introduced by CRISPR/Cas9, Choi et al. demonstrated the packaging of the Cas9 protein into VLPs for transient Cas9 exposure in cells [87]. The *Cas9* gene was fused to the *Gag* gene in a nonviral Gag/Pol expression vector. VLPs were produced by co-transfection with wild-type Gag/Pol and VSV-G plasmids. The protease cleavage site between Cas9 and Gag in the fusion plasmid allowed the release of the Cas9 protein during viral maturation [87]. Importantly, VLPs preloaded with Cas9 reduced off-target effects, as compared with LVs expressing Cas9 after integration into genomes [87], suggesting that transient Cas9 exposure with VLPs is advantageous. This is the first study to demonstrate the packaging of Cas9 into VLPs for gene editing, although it did not show the preloading of sgRNAs to Cas9 to form RNPs before delivery [87]. 

Mangeot et al. later developed this approach for gene editing with nanoblades, which are all-in-one VLPs preloaded with Cas9 and sgRNAs [88]. With this strategy, donor templates can also be loaded in VLPs for HDR [88]. Nanoblades were produced by the co-transfection of the retroviral murine leukemia virus Gag-Cas9 expression plasmid with the wild-type Gag-Pol, sgRNA, VSV-G, and baboon envelope plasmids [88]. Nanoblade-mediated gene editing was demonstrated in vitro in primary cells and induced pluripotent stem cells (iPSCs), as well as in vivo in mice [88]. The possibility of loading different sgRNAs into each nanoblade particle also suggested that gene editing is multiplexible [88]. The results of this study have opened the door for VLP-mediated gene editing in vitro and in vivo. Compared to vesicle production, the wild-type Gag-Pol plasmid is required in VLP production in order to provide protease to release Cas9. However, the competition between wild-type Gag and Gag-Cas9 proteins during VLP packaging restricts the dosage of the Cas9 protein that can be delivered [93]. Moreover, protease cleavage at the cryptic sites present in Cas9 may occur, resulting in its degradation [98]. 

Several studies demonstrated the production of vesicles pre-loaded with Cas9 RNPs for gene editing [89,90]. Campbell et al. used the Takara Guide-it CRISPR/Cas9 Gesicle Production System to produce gene editing vesicles, which are called gesicles, through the overexpression of VSV-G and the interaction between Cherry Picker red proteins and Cas9 RNPs [89]. In contrast, Montagna et al. produced gene editing vesicles, which they termed VEsiCas9, by the co-transfection of the HIV-1 Gag-Cas9 expression plasmid with sgRNA and VSV-G plasmids [90]. The production in both studies used the passive incorporation of Cas9 into the vesicles [89,90]. Recently, Gee et al. developed a robust production system of active Cas9 incorporation into vesicles, which they termed NanoMEDIC [91]. Cas9 incorporation into a vesicle was induced by the addition of a ligand (AP21967) to trigger the specific interaction between the FRB and FKBP12 domains [91]. In addition to gene editing, Gee et al. demonstrated that NanoMEDIC successfully induced exon skipping in vitro and in vivo in different models of Duchenne muscular dystrophy [91]. These results further demonstrated the applicability of vesicles for in vivo therapy. 

Taken together, these studies demonstrate the superior safety features of vesicle-mediated Cas9 RNP delivery, which do not integrate into the genome and provide transient Cas9 exposure [89,90,91]. Montagna et al. and Gee et al. also demonstrated the possibility of multiplexed gene editing with vesicles [89,90]. Because protease cleavage is not required in vesicle production, the vesicle system is not hampered by protease-mediated protein degradation or the competition between wild-type Gag and Gag-Cas9 proteins during packaging, which are the limitations in the VLP-based system. However, no data are currently available for the successful packaging of donor templates into vesicles for HDR-mediated gene editing. Further investigation is warranted to develop complete all-in-one preloaded vesicles for gene editing.

## 5. Conclusions and Future Perspectives

CRISPR/Cas9 gene editing technology is well-developed for in vitro applications. Without safety concerns in patients, most of the delivery strategies are suitable options for efficient editing. The off-target effects due to long-term Cas9 exposure are the biggest challenges for in vitro experiments. LV transduction results in permanent Cas9 expression, which increases the likelihood of off-target effects over that of other methods that impart transient Cas9 expression [9,87]. The delivery of plasmid DNA expressing Cas9 is more prone to off-target effects than is the direct delivery of the Cas9 protein by physical or chemical methods [7,8]. Longer Cas9 exposures to genomes are more susceptible to off-target cleavage [9,87]. When a transient system is used to reduce the duration of Cas9 exposure, the editing specificity of Cas9 is increased [9,87]. Because the EV-based delivery of Cas9 RNPs is transient, it outperforms many delivery methods by reducing off-target effects. Moreover, cell viability is not affected after transduction by EVs, but high levels of cell death are observed after electroporation. EVs also have the efficiency of viral systems. Therefore, EV-based systems are generally more efficient, and require less optimization, than do chemical-based systems. 

The delivery of CRISPR/Cas9 is also rapidly progressing in ex vivo applications. The treatment of hematopoietic diseases is made possible by harvesting patient hematopoietic stem cells for ex vivo modification before the autologous transplantation of the edited/modified cells back into patients [99]. However, the cost and effort to harvest the stem cells from each patient for autologous transplants negate the benefits of ex vivo cell therapy. Therefore, iPSCs have quickly become a popular cell platform for gene editing research [100], and which provide an unlimited supply of materials for gene editing and can be generated from easily accessible cell types, such as fibroblasts and peripheral blood cells [100]. Recently, the idea of universal donor cells has directed even more attention to the therapeutic values of iPSCs. By knocking out human leukocyte antigen class I and II in iPSCs, the cell products generated after differentiation are considered “off the shelf” and compatible with all patients [101,102]. This will streamline the process of ex vivo cell therapies. LVs and electroporation are commonly used to deliver CRISPR/Cas9 ex vivo [103], but LVs have safety issues and electroporation causes high levels of cell death. In the future, further development of EV-based delivery systems will offer a safe and efficient gene editing in ex vivo cell therapy. 

To date, the CRISPR/Cas9 system is not widely used in clinical trials. The trials that have investigated CRISPR/Cas9 gene editing to date only do so at the ex vivo level, by either modifying stem cells or T cells before transplanting them back into patients (ClinicalTrials.gov). Most current Cas9 delivery methods either have safety issues or low efficiency that preclude them from in vivo applications in patients. Based on the results from recent studies, EVs offer a transient, multiplexible, and all-in-one delivery platform for gene editing. Non-integrating EVs have no risk of insertional mutagenesis. The transient exposure of Cas9 to the cells dramatically reduces the chance of off-target effects. However, all of these studies used ultracentrifugation to concentrate EVs [87,88,89,90], which is not a scalable method or compatible with good manufacturing practice settings. Further investigation using scalable methods of purification and concentration, such as fast protein liquid chromatography, is needed. Because the genome quantitation of the titers of EVs is not possible with PCR-based assays, these studies used Western blots or dot blots to quantify the amount of Cas9 protein in EVs [87,88,89,90]. However, these methods may not be sensitive enough to detect low concentrations of Cas9. Moreover, the amount of Cas9 protein detected, which varies by the number of Cas9 protein copies packaged into each EV particle, does not reflect the actual titers of EVs produced. Without knowing the actual yield of EVs, the optimization of their production is difficult. Therefore, a precise method for EV quantitation is needed. Furthermore, EV-based systems have not yet been demonstrated to deliver base editors for gene editing. Because base editing and prime editing are safer approaches than conventional editing, in terms of genotoxicity, investigation into the possibility of their delivery by EVs is crucial. Taken together, CRISPR/Cas9-mediated gene editing is evolving rapidly, including its delivery methods. The EV-based system offers a safe and transient method to deliver Cas9 in vitro and in vivo. Further development and the optimization of this delivery platform will open the door for CRISPR/Cas9 gene editing in future clinical trials.

## Figures and Tables

**Table 1:** Comparison of common clustered regularly interspaced short palindromic repeats (CRISPR)/Cas9 delivery strategies.

Strategy	Viral Delivery				Non-Viral Delivery				
	LV	AAV	AV	EV	Microinjection	Electroporation	Cell Penetrating Peptide	Lipid-Based Nanoparticle	Gold Nanoparticle
**Cas9 Delivery Format**	*DNA*	*DNA*	*DNA*	Protein	*DNA*, *mRNA* or protein	*DNA*, *mRNA* or protein	Protein	*DNA*, *mRNA* or protein	Protein
**Delivery Efficiency**	+++	++	++	++	+	+++	+	+	++
**Safety Concern**	+++	+	++	+	+	+	+	+	+
**Cost**	+	++	++	+	+++	+++	+	+	++
**Technical Requirement**	+	++	+++	+	+++	+	++	+	++
**Major Advantages**	Efficient delivery; Large cloning capacity	Non-integrating	Non-integrating	Non-integrating; transient exposure; multiplexible; all-in one format	Direct delivery; Dosage more controllable	Efficient delivery; Easy to operate	No risk of virus	FDA-approved; Low stress to the cells	No risk of virus
**Major Limitations**	Random integration; Insertional mutagenesis	Limited cloning capacity	immune response	Limited quantification method	Technical challenging; in vivo work not feasible	Cell viability issue; in vivo work difficult	Variable efficiency depends on cell types; requires extensive optimization		
**Major Applications**	in vitro and ex vivo	in vivo	in vivo	in vitro, ex vivo and in vivo	in vitro and ex vivo	in vitro and ex vivo	in vitro and in vivo	in vitro and in vivo	in vitro and in vivo

AV, adenovirus; AAV, adeno-associated virus; EV, extracellular vesicle; LV, lentivirus; + denotes low; ++ denotes medium; +++ denotes high.
